# Supplementary material for: Comparing UNet configurations for anthropogenic geomorphic feature extraction from land surface parameters
Source: PLoS One. 2025 Jun 10;20(6):e0325904. doi: 10.1371/journal.pone.0325904 (PMC12151443; doi:10.1371/journal.pone.0325904)
Supplement: Attachment — Submitted filename: response_to_reviewers.pdf [file pone.0325904.s002.pdf]

RESPONSE TO REVIEWERS

OVERVIEW

Thanks for reviewing our manuscript. Below, the reviewers' comments are provided in **BOLD BLACK** text. Our comments are provided in red text. Sections extracted from the paper are *red and italicized*. We have tried our best to address the reviewers' comments. We are willing to make additional edits if necessary.

REVIEWERS QUESTIONS

1. Is the manuscript technically sound, and do the data support the conclusions?

Reviewer #1: Yes
Reviewer #2: Yes

2. Has the statistical analysis been performed appropriately and rigorously?

Reviewer #1: Yes
Reviewer #2: Yes

3. Have the authors made all data underlying the findings in their manuscript fully available?

Reviewer #1: No
Reviewer #2: Yes

The three example datasets used in this study (mineBenchDL, terraceDL, and vfillDL) have been made freely available via FigShare under a CC BY 4.0 license. The geodl R package source code is available on GitHub (https://github.com/maxwell-geospatial/geodl). The authors of this paper generated this R package. The package is also available on the Comprehensive R Archive Network (CRAN) (https://cran.r-project.org/web/packages/geodl/index.html). We hope this addresses your concern.

4. Is the manuscript presented in an intelligible fashion and written in standard English?

Reviewer #1: Yes
Reviewer #2: Yes

REVIEWERS COMMENTS

REVIEWER 1

The research objective of this paper is to compare the impact of different UNet architecture modifications on geomorphological feature extraction, in particular their performance under different amounts of training data. The authors used three different datasets: agricultural terraces, mining steps, and valley fill surfaces, and tested a variety of UNet variants, including activation function substitution, residual concatenation, dilated convolution, SE modules, attention gates, and MobileNetV2 encoders. The experimental design covers different training sample sizes (50 to full data) and evaluates various performance metrics. Among them, the following elements need further modification.

Thanks for reviewing our manuscript.

The literature review section may not be in-depth enough, especially regarding the application of UNet variants in other domains, have similar studies been conducted and have the authors adequately cited the relevant literature?

Thanks for this comment. We have expanded the first section of the Background section focused on the UNet architecture as follows. We feel that this now provides a better framing of the architecture and its applications.

UNet is widely used in geospatial and remote sensing applications due to its ability to capture fine-scale spatial patterns and maintain high segmentation accuracy across diverse landscapes. For example, Redoloza et al. [40] successfully used UNet to delineate subsurface agricultural drainage from satellite imagery, demonstrating its capability in agricultural landscapes. Dui et al. [41] applied an improved UNet model for automatic detection of photovoltaic facilities from Sentinel-2 MSI imagery, highlighting its effectiveness in multispectral segmentation. In urban studies, Aghayari et al. [42] and Zhao et al. [43] employed UNet-based architectures for building extraction from aerial imagery, emphasizing its robustness to varying object characteristics in remotely sensed data. In ecological applications, Wagner et al. [44] used UNet to map canopy palms in the Amazon rainforest, showcasing its strength in identifying vegetation patterns from high-resolution imagery. Jiao et al. [45] refined UNet for cloud and shadow segmentation in Landsat imagery, addressing limitations in traditional cloud detection techniques. Additionally, Abdollahi et al. [46] leveraged UNet for road segmentation from aerial imagery, further illustrating its adaptability in geospatial feature extraction. Despite these varied applications, relatively few studies have systematically compared multiple UNet variants across tasks within a unified framework. This study aims to fill that gap by evaluating key architectural modifications for geomorphic feature segmentation.

The experimental part, although testing a variety of variants, lacks discussion on model complexity and computational efficiency, such as training time and resource consumption of different variants, which is important for selection in practical applications.

Thanks for this comment. In the Methods section, and specifically in the Training Process subsection, we have now included (1) the number of trainable parameters, (2) the number of floating-point operations (FLOPS), and (3) the number of multiply-add operations (MACs). These are provided in Table 3, which is new. Note that the counts of trainable parameters were originally included in Table 2 but have now been moved to the new Table 3. The FLOPs and MACs were not included in the prior draft.

Table 3. *Model complexity and computational cost comparison. 1 GFLOP = 1 billion FLOPs; 1 GMAC = 1 billion MACs.*

Model/Augmentation Name	***Trainable Parameters***	***FLOPs (GFLOPs)***	***MACs (GMACs)***
Base UNet	*2,315,186*	*303.58*	*150.87*
Leaky ReLU Activation	*2,315,186*	*303.58*	*150.87*
Swish Activation	*2,315,186*	*303.58*	*150.87*
Residual Blocks	*2,426,242*	*323.26*	*160.39*
DC Bottleneck	*1,542,562*	*287.76*	*142.96*
Squeeze and Excitation	*2,320,626*	*303.66*	*150.87*
Attention Gates	*2,382,158*	*307.79*	*152.9*
MobileUNet	*6,459,578*	*307.07*	*151.92*

We have also added a paragraph to accompany Table 3:

To compare models beyond accuracy, we considered computational complexity and model efficiency using standardized metrics: the number of floating-point operations (FLOPs), multiply-add operations (MACs), and total number of learnable parameters, as shown in Table 3. These measures offer architecture-specific insights into scalability and feasibility for practical deployment. While training and inference times are important in applied contexts, they were not used for direct comparison due to their sensitivity to implementation-specific variables such as hardware specifications, mini-batch size, and optimizer settings. By focusing on FLOPs, MACs, and parameter count, we provide a more controlled and reproducible basis for evaluating the computational efficiency of different UNet configurations for large-scale geomorphic feature mapping.

As this paragraph explains, we have reported and discussed trainable parameter counts, FLOPS, and MACs since computational time is impacted by many non-architecture specific factors.

We have also added to the Discussion section as follows:

In addition to segmentation performance, computational complexity is an important factor in evaluating the practicality of deep learning architectures for large-scale or resource-constrained applications. Table 3 presents a comparison of model complexity and computational cost across the UNet variants evaluated in this study. The number of trainable parameters varied considerably among models, ranging from 1.54 million for the DC Bottleneck model to 6.46 million for MobileUNet. In terms of computational cost, measured by FLOPs and MACs, the DC Bottleneck model was the most efficient, with the lowest FLOPs (287.76 GFLOPs) and MACs (142.96 GMACs). The DC Bottleneck variant is especially noteworthy for being both lightweight and computationally efficient while still performing competitively. Its lower resource demands make it particularly attractive for deployment in scenarios with limited computing resources. Conversely, the MobileUNet configuration exhibited the highest parameter count, nearly three times that of the base UNet, but with only modest increases in computational operations. This highlights MobileNetV2's depthwise separable convolutions, which effectively trade increased memory usage for more efficient computation. Additionally, while the residual blocks and attention modules introduced moderate increases in computational complexity, their use might still be justified if they lead to substantial improvements in model accuracy or feature delineation. Therefore, we emphasize that decisions about adopting complex modules should balance measurable performance gains against computational costs and resource availability.

The description of the datasets may not be detailed enough, e.g. does the number of samples in each dataset, the distribution of categories, feature differences, etc. affect the interpretation of the results?

Thanks for this comment. We have tried to further clarify the characteristics of the three datasets in our revision. We have added the following text to the Study Areas and Datasets subsection of the Methods section:

The characteristics of each dataset have important implications for model performance and interpretation of results. Terrace and mine bench features are typically long and narrow, resulting in a higher proportion of edge pixels within image chips and introducing challenges for segmentation, particularly in boundary delineation. In contrast, valley fill features tend to be larger and more compact, with greater interior area and fewer edge pixels. Additionally, the number of available training chips varied considerably across datasets (see Table 1), which may have influenced training dynamics, especially in experiments using limited sample sizes. These differences underscore the importance of considering both the spatial structure of features and the quantity of training data when interpreting segmentation outcomes and selecting appropriate UNet configurations.

We feel that this addition enhances content included in the original draft and aids the reader in understanding the unique characteristics of each task. From the original draft, Table 1 summarizes the number of training, validation and test chips used for all three datasets:

Table 1. *Overview of the available training, validation, and test chips for each dataset used.*

Dataset	*Terraces*	*Valley Fills*	*Mine Benches*

Training	*50, 100, 250, 500, 3981*	*50, 100, 250, 500, 1222*	*50, 100, 250, 500, 4164*
Validation	*1488*	*423*	*2334*
Test	*4183*	*423*	*2304*

Also, Figure 7 visualizes the three features of interest and their associated presentation in the land surface parameters used in the study along with the spatial partitioning of the training, validation, and test sets.

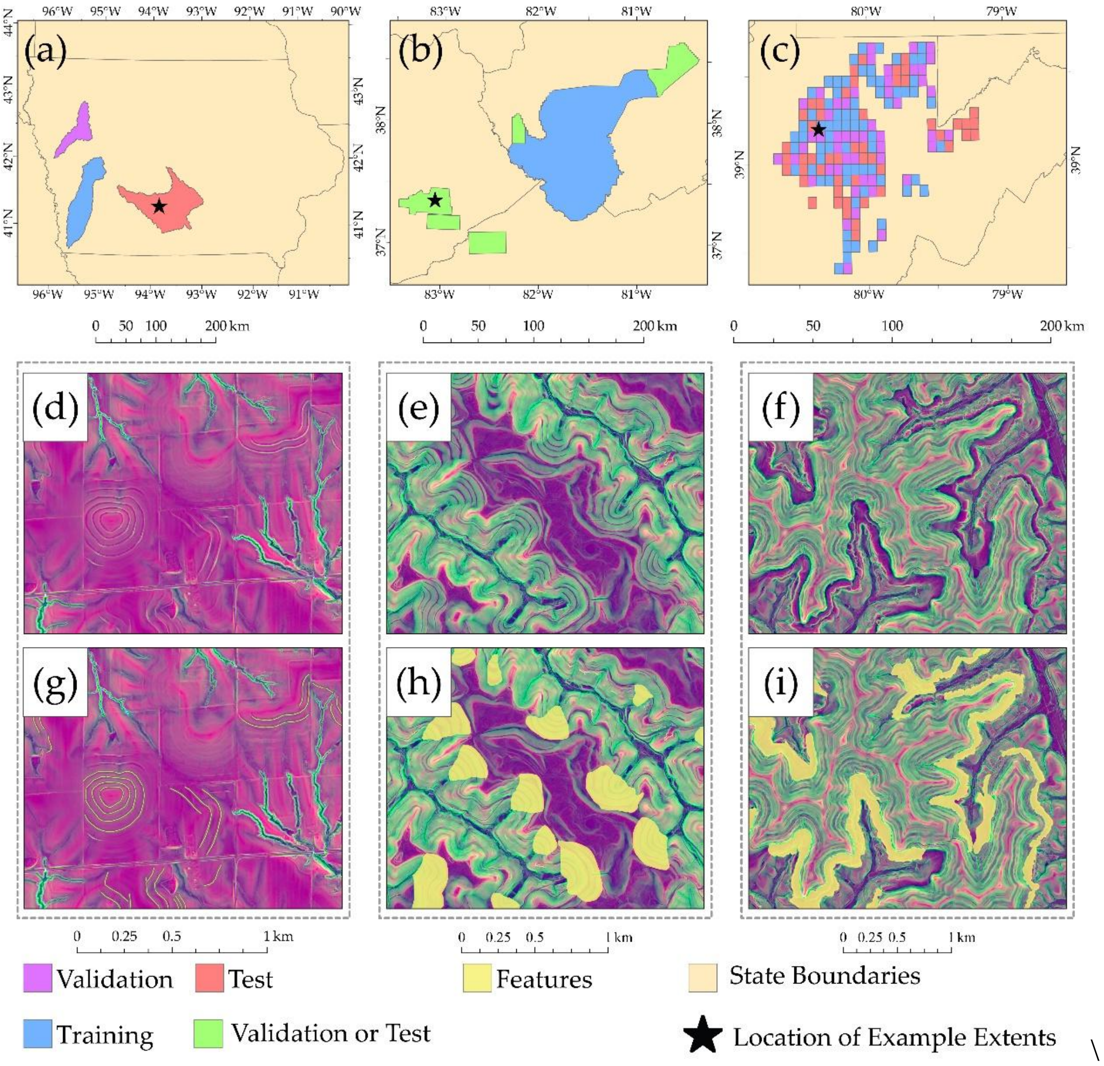

Figure 7. *Training, test, and validation areas for (a) agricultural terraces (terraceDL) dataset [29] in Iowa, USA; (b) surface coal mining valley fill faces (vfillDL) dataset [30] in southern West Virginia, eastern Kentucky, and southwestern Virginia, USA; and (c) historic mine benches (minebenchDL) dataset [31] in northern West Virginia, USA. (d) through (i) show example terrain surfaces and associated geomorphic features as examples.*

Lastly, the datasets are also described in the Study Areas and Datasets subsection of the Methods section as follows. We feel that the added paragraph, provided above, helps to better characterize the classification tasks following the general information provided in the prior paragraphs.

The data used in this study are described in Figure 7. The terraceDL dataset [29] (Figure 7(a), (d), and (g)) originated from the Iowa Best Management Practices (BMP) Mapping Project [74], where agricultural terrace data were generated as line vector features through manual digitization and interpretation of lidar-derived topographic data and aerial orthophotography. These terraces serve as intentional modifications to the landscape, aimed at mitigating agricultural-induced soil loss by impeding both sheet and rill erosion as well as gully formation. Figure 7(a) illustrates the geographic regions within Iowa, USA used for the study's training, validation, and testing phases, demarcated by hydrologic unit code (HUC) 8 watershed boundaries and containing 66,000, 15,505, and 26,453 terraces, respectively. Figure 7(d) and (g) show example features on the landscape. The terrain representation used in the figures are discussed below.

The vfillDL dataset [30] (Figures 7(b), (e), and (h)) was created by our lab group using manual digitizing and interpretation of digital terrain models and supplemental data. Valley fills are man-made landforms resulting from surface coal mine reclamation. Although the federally implemented Surface Mine Control and Reclamation Act (SMCRA) in the United States mandates restoring mine sites to their "approximate original contour," the steep terrain of southern West Virginia, eastern Kentucky, and southwestern Virginia makes this restoration impractical. Consequently, mining operations are allowed to modify the landscape by leveling mountaintops and depositing overburden material in nearby valleys, creating valley fills [75-78]. As illustrated in Figure 7(b), a large area within West Virginia, which contained 1,105 valley fills, was used to train the model. Neighboring regions in West Virginia, as well as areas in eastern Kentucky and southwestern Virginia, were combined and divided into two equal datasets. These datasets served as validation areas at the end of each training epoch and as the test set for final model evaluation. Collectively, these regions contained 1,178 valley fills.

The minebenchDL dataset [31] (Figures 7(c), (f), and (i)) was produced by the authors through on-screen digitizing and interpretation of topographic data derived from lidar, aerial orthophotography, and ancillary mine disturbance extents. Mine benches are remnants of historic surface coal mining. Prior to SMCRA, operators were not required to reclaim the landscape to "approximate original contour"; as a result, remnant benches are abundant in the Appalachian coalfields. During the mining process, coal seams were excavated along topographic contour, resulting in long, linear highwalls and associated benches that meander along topographic contour in areas of disturbance. Overburden rock material was often placed on the slopes beneath the benches, further modifying the terrain. Many of the mine sites have now been revegetated and are not obvious in aerial imagery. However, the benches are generally very distinctive in high spatial resolution digital terrain data. As highlighted in Figure 7(c), the data were partitioned into separate training, validation, and test sets based on 1:24,000 scale United States Geological Survey (USGS) quarter quadrangle boundaries. The 169 mapped quarter quadrangles were randomly partitioned such that 69 were used for training, 50 were used for validation, and 50 were used for testing. Only quarter quads that included mine bench features were used. Collectively, 2,416 mine benches occurred within the mapped extent.

In the discussion section, the authors mention that the model performance tends to stabilise after 500 samples, but the reason is not explored in depth, is it related to the model capacity or data characteristics?

Thanks for this comment. We have now added to the Discussion to address this point:

The observed stabilization of model performance beyond 500 training chips likely results from the combined effect of dataset characteristics, particularly the degree of visual separability of the features and their consistency, and the inherent capacity of the UNet architecture, which appears sufficient to capture key spatial patterns and generalize effectively beyond this training size. However, it is important to note that this may not extrapolate to other geomorphic feature extraction tasks due to feature characteristics, number of classes being differentiated, within class heterogeneity, and the degree of differentiation between the feature(s) of interest and the rest of the landscape.

In the conclusion, it is mentioned that MobileNetV2 performs poorly with a small amount of data, but it is not discussed whether it is because the pre-training weights are not used, and whether the effect of migration learning is considered?

Thanks for this comment. We have added to the Discussion to address this point:

3. *Complex backbone encoders, such as MobileNetV2, may require larger training sets to be successfully implemented due to their use of depthwise separable convolutions and linear bottlenecks, which demand more data to effectively learn nuanced spatial features particularly without pretraining. Larger architectures may also be more prone to overfitting, especially when the training set size is small and transfer learning is not implemented. Transfer learning was not explored in this study; however, it was assessed in our prior study, Maxwell et al. [98], where transfer learning was generally noted to be of value for geomorphic feature extraction tasks.*

REVIEWER 2

Thanks for reviewing our manuscript.

Your manuscript has made the main points of your study clear and the manuscript was well prepared. But my concerns are that if you look at the table 4 to 6, you may find there are a big difference between the OA and the F1-Score. Did you use the independent datasets that were different from the one for building the models and there was no strong spatial connection between some samples in model built datasets and some other samples in validation datasets? How do you explain this difference that OA is extremely high but F1 is extremely low?

Thanks for this comment.

The primary issue here is class imbalance. Since the features of interest (i.e., valley fill faces, agricultural terraces, or mine benches) make up a small percentage of the total landscape, the

overall accuracy is high since most cells/pixels in the predicted extents are from the background class and are correctly predicted to the background class. Thus, overall accuracy is, generally speaking, a poor assessment metric for these problems. Instead, F1-score, and the related precision and recall metrics, are much more meaningful/informative. Precision and recall offer insights into the model's ability to minimize commission and omission errors for the positive class, respectively. Relative to the positive case, or the geomorphic feature of interest, commission errors result from background pixels being incorrectly predicted as the geomorphic feature of interest while omission errors result from pixels occurring within the geomorphic feature of interest being incorrectly predicted to the background class. The F1-score, being the harmonic mean of precision and recall, provides a combined measure of the model's performance.

We have tried to clarify this point in the Results section:

A key observation is that models tended to stabilize when provided with 500 or more training samples, as improvements in F1-score, recall, and precision became more incremental. Although overall accuracies were consistently high across all experiments, this is largely due to class imbalance. A significant portion of the landscape is classified as the background class, which is generally well predicted, resulting in a large proportion of correctly mapped pixels. Consequently, overall accuracy can inflate perceived model performance even when the positive class is poorly segmented. Therefore, it is crucial to evaluate the models using F1-score, recall, and precision specifically for the positive cases.

In short, this issue arises from class imbalance, which makes overall accuracy an uninformative metric in this case. Note that we took great care to reduce spatial autocorrelation between the training, validation, and test splits by making use of a geographic stratification, as visualized in Figure 7(a), (b), and (c).

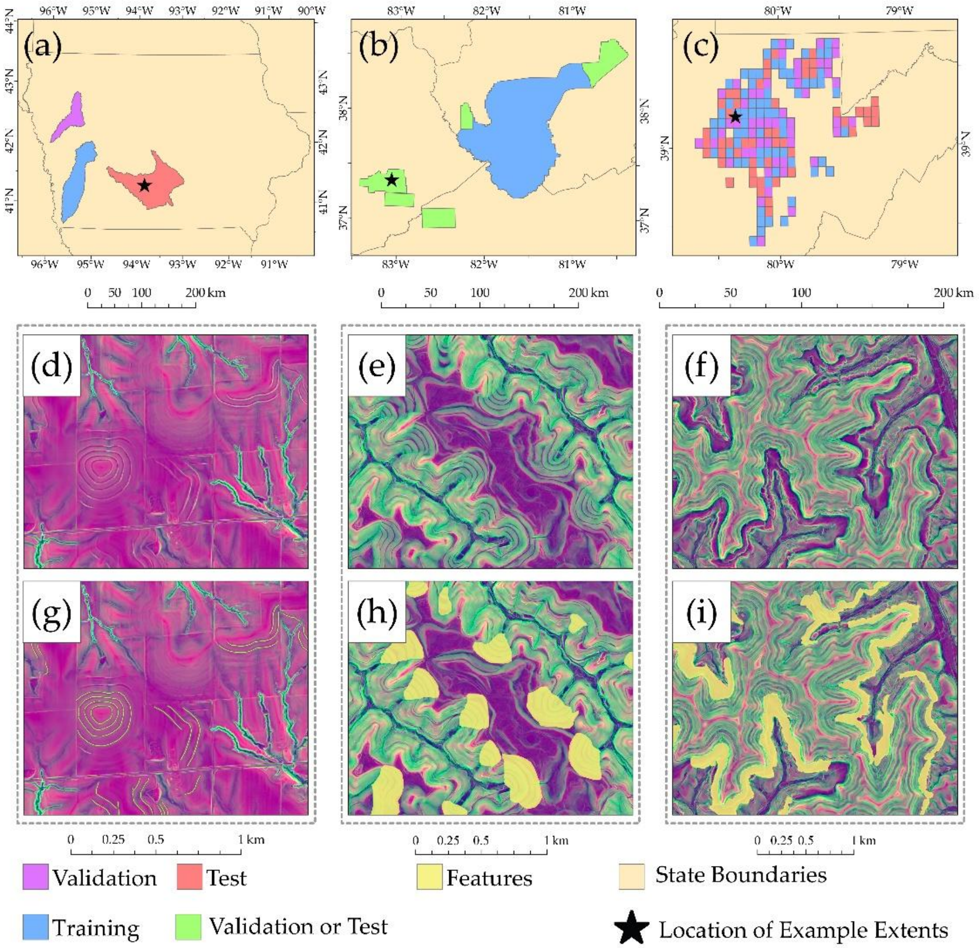

You still look at these tables from 4 to 6. You may also find the increment of OA was small for all configurations as the number of chips increased while the increment of F1 was a little bit high. What happened? Was it inappropriate to use OA to evaluate the accuracy of classified results? Why did you think the sample size of 500 is appropriate in the applications of Unet for classification?

Thanks for this comment.

Please see our response to your prior comment where we explain the issues with overall accuracy resulting from class imbalance.

We have also expanded the Discussion section to further discuss stabilization with samples sizes greater than or equal to 500.

The observed stabilization of model performance beyond 500 training chips likely results from the combined effect of dataset characteristics, particularly the degree of visual separability of the features and their consistency, and the inherent capacity of the UNet architecture, which appears sufficient to capture key spatial patterns and generalize effectively beyond this training size. However, it is important to note that this may not extrapolate to other geomorphic feature extraction tasks due to feature characteristics, number of classes being differentiated, within

class heterogeneity, and the degree of differentiation between the feature(s) of interest and the rest of the landscape.

By the way, check the line 512, the word ‘then’ was misspelled.

Thanks for catching this misspelling. This has been corrected.
